# Is Routine Omentectomy a Necessary Component of Cytoreductive Surgery and HIPEC?

**DOI:** 10.1245/s10434-022-12714-7

**Published:** 2022-10-28

**Authors:** Sohini Khan, Nguyen-Huong Doan, Mojgan Hosseini, Kaitlyn Kelly, Jula Veerapong, Andrew M. Lowy, Joel Baumgartner

**Affiliations:** 1 Division of Surgical Oncology, Department of Surgery, University of California San Diego, La Jolla, CA USA; 2 Department of Pathology, University of California San Diego, La Jolla, CA USA; 3 Department of Surgery, University of Wisconsin, Madison, WI USA

## Abstract

**Background:**

Cytoreductive surgery (CRS) with or without hyperthermic intraperitoneal chemotherapy (HIPEC) for peritoneal metastases traditionally includes omentectomy, even in the absence of visible omental metastases. We sought to determine the rate of occult histologic omental metastasis (OHOM), evaluate morbidity with omentectomy, and examine the rate of omental recurrence among patients undergoing CRS-HIPEC.

**Methods:**

All CRS-HIPEC procedures from August 2007 to August 2020 were included in this single-center, retrospective, cohort study. Procedures were divided into those that included greater omentectomy (OM) and those that did not (NOM). The incidence of OHOM was evaluated specifically among the OM group with a grossly normal omentum. Multivariate regression analyses were performed to evaluate return of bowel function, ileus, and morbidity in the OM and NOM groups.

**Results:**

Among 683 CRS-HIPEC procedures, 578 (84.6%) included omentectomy and 105 (15.4%) did not. The OM group had higher operative time, blood loss, peritoneal cancer index, number of visceral resections, and length of stay. In the OM group, 72 (12.5%) patients had a grossly normal omentum, and 23 (31.9%) of these had OHOM. Risk-adjusted return of bowel function, ileus, and 60-day complications were no different in the OM and NOM groups. Among 43 patients with residual omentum, 24 (55.8%) recurred, including 9 (20.9%) with omental recurrence.

**Conclusions:**

Histologically occult metastasis was present in one-third of patients undergoing omentectomy during CRS-HIPEC. Omentectomy did not increase the rate of overall morbidity, and one-fifth of patients with residual omentum later developed omental recurrence. Thus, omentectomy is warranted in the absence of gross metastases during CRS-HIPEC.

Cytoreductive surgery (CRS) and hyperthermic intraperitoneal chemotherapy (HIPEC) is used to treat patients with peritoneal mesothelioma and those with isolated peritoneal metastases secondary to gastric, appendiceal, colorectal, and ovarian primary malignancies.^1–5^ The goal of CRS is to resect all gross peritoneal metastases and may be followed by the administration of HIPEC with the intent to eliminate residual microscopic peritoneal metastases. There is no standardized CRS-HIPEC protocol for any given malignancy, and variations exist among institutions.^6^ Peritoneal recurrence rates vary by histology and disease burden but are primarily dependent on the completeness of cytoreduction.^7,8^

Greater omentectomy is typically performed during CRS to remove omental metastases, which are commonly found in patients with peritoneal metastases.^9,10^ Omentectomy has traditionally been performed even in the absence of visible omental metastases.^11^ However, the rate of omental metastases in the absence of visible disease is largely unknown, although it has been estimated to be around 15–20%.^12–14^ While the omentum has a physiologically uncertain role, there are potential risks of omentectomy, including injury to nearby structures and bleeding. Some have questioned the necessity of omentectomy for other surgical oncology procedures.^15^

The primary goal of this study was to determine the rate of occult omental metastases in patients undergoing CRS-HIPEC for peritoneal surface malignancy. The secondary goals were to determine the rate of complications in those undergoing omentectomy as well as to determine the rate of omental recurrence in those patients who did not undergo omentectomy.

## Methods

### Design and Eligibility

This was an institutional review board-approved, single center, retrospective, cohort study of all patients undergoing CRS-HIPEC at the University of California, San Diego (UCSD) from August 2007 to August 2020. All patients who underwent CRS-HIPEC during the operative period were included. The primary goal was to determine the incidence of occult histologic metastases in resected grossly normal greater omentum. Secondary goals were to compare the recovery in those who underwent omentectomy versus those who did not, as well as to determine the rate of recurrence in those with a residual omentum.

### Operative Details

All patients underwent CRS followed immediately by HIPEC per the standardized technique performed at UCSD.^16^ The extent of peritoneal metastases was recorded at time of surgery according to the Peritoneal Cancer Index (PCI),^17^ and the completeness of cytoreduction score (CC-score) was used to assess residual, unresected disease.^18^ HIPEC was performed after CRS using a closed-abdomen perfusion technique with 3–6 L, warmed perfusate and intraperitoneal chemoperfusion for 90 min with a goal intraperitoneal hyperthermia of 42 °C. Patients with appendiceal, colorectal, and small-bowel primary tumors were treated with 10 mg/L of perfusate of intraperitoneal mitomycin C. Patients with mesothelioma were administered 50 mg/m^2^ of cisplatin and 15 mg/m^2^ of doxorubicin. Patients with ovarian cancer were given 100 mg/m^2^ of cisplatin, and those with gastric cancer were given 50 mg/m^2^ of cisplatin and 10 mg/L of perfusate of mitomycin C. Visceral resections were defined as colon resection, small-bowel resection, appendectomy, anatomic hepatic resection, pancreatectomy, cholecystectomy, hysterectomy and/or oophorectomy, partial or total gastrectomy, or splenectomy. In patients undergoing an omentectomy, as much of the gastrosplenic ligament as feasible was removed if the spleen was not removed.

### Pathologic Evaluation

All operative specimens, including submitted greater omental specimens, were evaluated grossly and microscopically by using hematoxylin and eosin-stained slides by gastrointestinal and gynecological pathologists. Appropriate CAP (College of American Pathology) cancer and biomarker synoptic reporting system was used in diagnosis and staging of all the cases.

### Data Collection

Data were collected by using a prospectively maintained, peritoneal surface malignancy REDCap database and electronic medical record review. Baseline variables included age, primary tumor site, histology, operative (OR) time, estimated blood loss (EBL), PCI, CC-score, number of anastomoses and visceral resections, and length of stay (LOS). Appendiceal tumors were classified as low grade, which included low-grade appendiceal mucinous neoplasms and low-grade mucinous carcinomatosis peritonei, and high-grade tumors, which included high-grade appendiceal mucinous neoplasms, adenocarcinomas, and signet ring cell carcinomas. Procedures were divided into groups of those that included documentation of greater omentectomy (OM) and those that did not (NOM). A comparison was done of the pathology reports and the operative findings for omentectomy patients to discern which cases had grossly normal omentum and if there were occult histologic omental metastases (OHOM) or abnormalities in these specimens. For completeness, we included histologic identification of malignant cells as well as acellular mucin in the OHOM group. Patients who did not have an omentectomy and had a residual omentum were analyzed for the rate of recurrence, both within the omentum as well as extra-omental recurrences. We did not include patients in the omental recurrence analysis if it was unclear whether or not they had a retained omentum after surgery, as some patients already had a complete or partial omentectomy before undergoing CRS-HIPEC.

### Statistics

Chi-square analyses were performed for univariate categorical variables, logistic regression analyses were performed for covariant categorical variables, and Student’s *t*-test was used to compare continuous variables. The 60-day comprehensive complication index between patients who had omentectomy and those who did not also were analyzed using multiple linear regression, with adjustment for confounding variables. Days to bowel function was defined as the time to both upper (tolerance of solid food) and lower (first bowel movement) gastrointestinal function.

## Results

This study included 683 CRS-HIPEC procedures performed in 654 patients. Greater omentectomy was performed in 580 CRS-HIPEC procedures (84.9%, OM group), and it was not performed in 103 (15.1%, NOM group). There was a comparable ethnicity distribution between the OM and NOM groups—Caucasian: 248 (65.1%) versus 37 (71.2%); Hispanic: 75 (19.7%) versus 4 (7.7%); African American: 10 (2.6%) versus 1 (1.9%); Asian: 33 (8.7%) versus 6 (11.5%); other: 15 (3.9%) versus 4 (7.7%). The OM and NOM groups were of similar age and histology, but the OM group had higher PCI, EBL, number of visceral resections, and longer operative time than the NOM group (14 vs. 7, 200 cc vs. 100 cc, 2 vs. 1, and 442 min vs. 361 min, respectively; Table 1). The OM group also had a slightly higher proportion of CC-0/1 cytoreductions and longer LOS than the NOM group (97.3% vs. 92.2%, 9 days vs. 7 days, respectively). The rate of splenectomy was 36.4% in the OM group compared with 10.7% in the NOM group (*p* < 0.001).

**Table 1:** Baseline and operative details

Variable	Total (n = 683) n (%)/median (range)	Omentectomy (n = 580) n (%)/median (range)	No omentectomy (n = 103) n (%)/median (range)	*p* value
Age (yr)	54 (20–86)	55 (20–86)	52 (26–78)	0.153^e^
Primary/histology				0.484^f^
Appendix – LG^a^	252 (36.9)	220 (37.9)	32 (31.1)	
Appendix – HG^b^	169 (24.7)	140 (24.1)	29 (28.2)	
Colorectal cancer	142 (20.8)	114 (19.7)	28 (27.2)	
Mesothelioma^c^	62 (9.1)	56 (9.7)	6 (5.9)	
Ovary	29 (4.2)	25 (4.3)	4 (3.9)	
Small bowel	9 (1.3)	8 (1.4)	1 (1.0)	
Gastric	6 (0.9)	5 (0.9)	1 (1.0)	
Other^d^	14 (2.0)	12 (2.1)	2 (1.9)	
OR time (min)	429 (194–935)	442 (194–935)	361 (194–876)	**< 0.001**^e^
EBL	150 (0–4000)	200 (5–3500)	100 (0–4000)	**0.013**^**e**^
PCI	13 (0–36)	14 (0–36)	7 (0–25)	**< 0.001**^e^
CC-score				**0.039**^f^
CC-0	532 (77.9)	451 (77.8)	81 (78.6)	
CC-1	127 (18.6)	113 (19.5)	14 (13.6)	
CC-2/3	24 (3.5)	16 (2.8)	8 (7.8)	
No. anastomoses	1 (0–6)	1 (0–4)	1 (0–6)	0.079^e^
No. visceral resections	2 (0–10)	2 (0–7)	1 (0–10)	**< 0.001**^e^
LOS (days)	9 (3–68)	9 (3–68)	7 (3–34)	**0.024**^e^

*LG* low grade; *HG* high grade; *EBL* estimated blood loss; *PCI* peritoneal cancer index; *CC* completeness of cytoreduction; *LOS* length of stay

^a^Includes low-grade appendiceal mucinous neoplasms and low-grade mucinous carcinoma peritonei

^b^Includes high-grade appendiceal mucinous neoplasms, adenocarcinoma, and signet ring cell carcinoma

^c^Includes malignant mesothelioma (n = 56), well-differentiated papillary mesothelioma (n = 4), and multicystic mesothelioma (n = 2)

^d^Includes unknown primary (n = 4), desmoplastic small round cell tumor (n = 4), gallbladder carcinoma/cholangiocarcinoma (n = 2), pancreatic adenocarcinoma (n = 2), testicular cancer (n = 1), and urachal carcinoma (n = 1)

^e^Student’s *t*-test

^f^Chi-square test

The OM group had a similar time to return of bowel function (time to first bowel movement and tolerance of solid diet), postoperative ileus rate, and 60-day comprehensive complication index compared with the NOM group, when adjusted for OR time, EBL, PCI, and number of visceral resections (7.4 days vs. 6.1 days, 12.8% vs. 11.7%, 17.1 vs. 15.0, respectively; Table 2). There was no difference in the rate of postoperative emesis in the OM and NOM groups (31.7% vs. 25.5%, respectively, *p* = 0.23). If a gastric tube was placed intraoperatively for gastric decompression, the OM group did not have significantly more total output throughout the admission than the NOM group (median 4.6 L vs. 3.9 L, respectively, *p* = 0.18). If a nasogastric tube was placed intraoperatively instead of a gastric tube, it was removed on median POD 2 in the OM and NOM groups (*p* = 0.39).

**Table 2:** Omentectomy and morbidity

Variable	Omentectomy	No Omentectomy	*p* value^c^
Bowel function return (days)^a^	7.4	6.1	0.426
Postoperative ileus^b^	12.8%	11.7%	0.710
60-day Comprehensive Complication Index	17.1	15.0	0.733

^a^Time to first bowel movement and tolerance of solid food

^b^>7 days before first flatus or bowel movement

^c^By linear/logistic regression adjusted for operating room time, EBL, PCI, and number of visceral resections

**Fig. 1:**
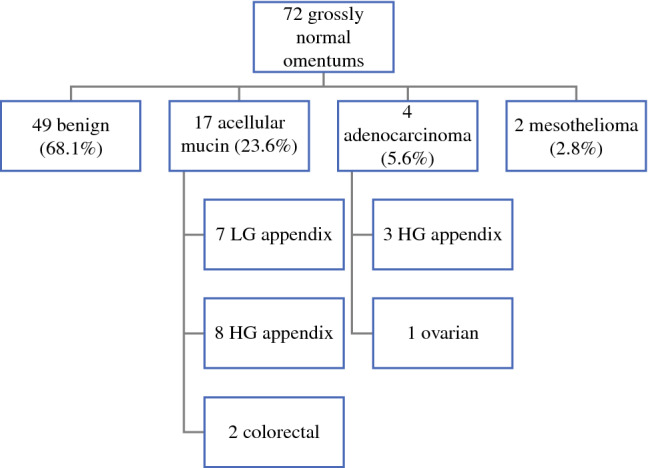
Grossly normal omentum histology and primary tumor sites. *LG* low grade; *HG* high grade

Among the OM group (n = 580), 452 patients (77.9%) had gross intraoperative evidence of omental metastases, whereas 72 (12.4%) had a grossly normal omentum, and 56 (9.7%) cases had no documentation of gross omental appearance. Of the 452 patients with gross intraoperative omental metastasis, there was histologic confirmation of omental metastasis in 421 (93.1%). There were a total of 72 cases with a grossly normal omentum intraoperatively. On final pathologic assessment of these 72 cases, 49 (68.1%) were determined to have benign histology and 23 (31.9%) had OHOM (Fig. 1). The majority of the OHOM cases had acellular mucin (n = 17, 23.6% of all grossly normal omentums), four (5.6%) had adenocarcinoma, and two (2.8%) had mesothelioma. The mean PCI of those with OHOM was higher than those with a histologically benign omentum (9 vs. 6, *p* = 0.013).

Among CRS-HIPEC procedures for low-grade appendiceal malignancies with grossly normal omentectomy (n = 22), seven (31.8%) had OHOM (all with acellular mucin). Eleven of 24 (45.8%) patients with high-grade appendiceal malignancies (8 with acellular mucin, 2 with adenocarcinoma), 2 of 18 (11.1%) with colorectal cancer (both with acellular mucin), 2 of 3 (66.7%) with mesothelioma, and 1 of 3 (33.3%) with ovarian carcinoma (with adenocarcinoma) had OHOM.

**Fig. 2:**
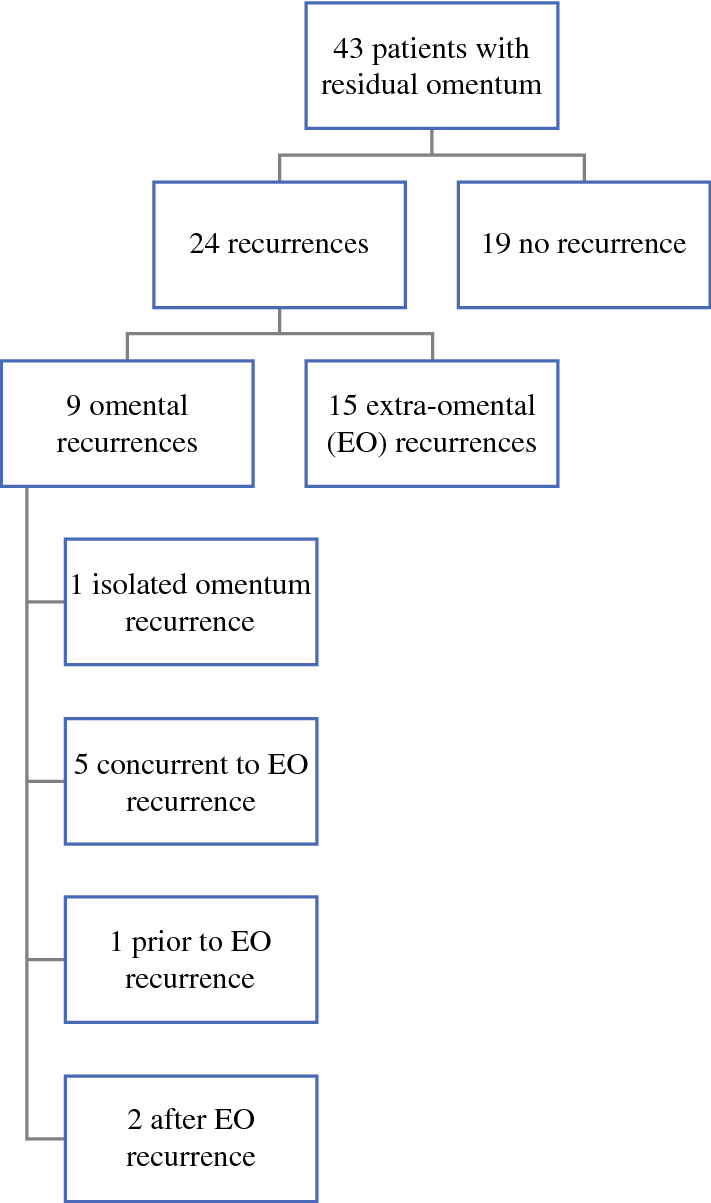
Chronology of omental recurrence. *EO* extra-omental

Forty-three cases identified in the NOM group had a residual omentum described in the operative note. Of these patients, 24 (55.8%) developed recurrent disease after a median follow-up of 25.9 months (range: 0.2–147.6; Fig. 2). Nine of these recurrences (37.5%) occurred in the residual omentum, and 15 (62.5%) had extra-omental recurrence. Of those with omental recurrence, there was only one patient who had an isolated recurrence in the omentum, and the remainder also had extra-omental recurrence.

## Discussion

In this series of 683 consecutive CRS-HIPEC procedures, we performed omentectomy in approximately 85% of cases. Approximately 75% of omentectomies had gross omental metastases. Among those with excision of a grossly normal omentum, OHOM was present in more than one-third of patients. This is slightly higher than the rate reported in other smaller series (15–20%).^12–14^ It is unclear whether acellular mucin was included as OHOM in other series, as it was in our study, in order to maximize inclusion of histologic abnormalities. This difference may explain the higher rate of OHOM in the present study, as acellular mucin accounted for the majority of OHOM in our series. The clinical significance of acellular mucin in specimens submitted during CRS-HIPEC is unclear. While by definition, acellular mucin itself is benign, it may indicate the presence of nearby neoplastic mucin-producing cells, which can be missed on pathologic evaluation if infrequently distributed in the specimen, and some patients with resected peritoneal acellular mucin for low-grade appendiceal mucinous neoplasms have been known to recur.^8,19,20^ If the acellular mucin cases are excluded from the OHOM group in our study, we still identified approximately 10% of grossly normal omentums with histological omental metastases.

Omentectomy also did not appear to increase the rate of overall morbidity, as the time of return of bowel function, ileus, or overall 60-day Comprehensive Complication Index was no different in the omentectomy group versus those without omentectomy, after adjusting for OR time, EBL, PCI, and the number of visceral resections. As a surrogate for delayed gastric emptying, we noted there to be no difference in the rate of emesis, gastric tube output, or length of time a nasogastric tube was used for gastric decompression between the two groups. We typically attempt to preserve the gastroepiploic vascular arcade during omentectomy unless it is directly invaded by tumor. This may have contributed to reduced morbidity including delayed gastric emptying in the omentectomy group; however, a randomized, controlled trial of gastroepiploic artery preservation with omentectomy during CRS-HIPEC found no difference in delayed gastric emptying in those with gastroepiploic artery resection, suggesting an alternate etiology of delayed return of bowel function in this population.^21^

Among those with documented retained omentum after CRS-HIPEC, we found that approximately half of these patients recurred, and more than one-third of these patients developed recurrence within the omentum. However, only one patient had evidence of isolated omental recurrence without extra-omental recurrence. The clinical significance of omental recurrence is unclear, and it may serve as a marker of recurrent disease rather than a mediator of further recurrence. However, given the goal of cytoreductive surgery is resection of all disease, we believe that routine omentectomy is reasonable.

This study has several limitations. It is a retrospective, single-institution study, and despite careful medical record review, data collection was potentially limited by incomplete documentation of prior surgical procedures, operative description of gross omental appearance, and follow-up. We only included patients in the retained omentum group where it was clear from the medical record that the omentum was retained. As such, it is possible that some patients were excluded who still had an omentum or portion of omentum remaining, distorting the true proportion of patients who had recurrent omental metastasis. Furthermore, performing an omentectomy did not necessitate a splenectomy; although as much of the gastrosplenic ligament was removed in patients undergoing omentectomy without splenectomy, there may have been some residual omentum left behind which was a potential source for omental recurrence. There also is the possibility of sampling error from the pathologist, because the entire omentum was not sectioned in most cases, potentially missing occult disease, as well as the possibility of underreporting of gross omental disease by the surgeon. Imaging-based documentation of peritoneal recurrence also is known to be limited in its accuracy, possibly leading to inaccurate reporting of omental-based recurrences. However, our series was performed at a peritoneal surface malignancy referral institution by high-volume surgeons and pathologists, minimizing the variability between cases. The rationale for why the omentum was left in some patients was at the surgeon’s discretion, but most often it was left due to a low burden of disease in cases without gross evidence of omental metastasis (as indicated by the lower PCI score in this group).

## Conclusions

Patients undergoing CRS-HIPEC with greater omentectomy, who had a grossly normal omentum, had a high rate of occult histologic omental metastasis. Omentectomy was not associated with higher morbidity than patients who did not have an omentectomy. Additionally, one-fifth of patients who did not have an omentectomy had omental recurrence. Routine omentectomy in the absence of gross metastasis therefore is recommended during CRS-HIPEC procedures.
